# Phenotypic and functional evaluations of peripheral blood monocytes from chronic-form paracoccidioidomycosis patients before and after treatment

**DOI:** 10.1186/s12879-014-0552-x

**Published:** 2014-10-16

**Authors:** James Venturini, Ricardo Souza Cavalcante, Márjorie de Assis Golim, Camila Martins Marchetti, Priscila Zacarias de Azevedo, Bárbara Casella Amorim, Maria Sueli Parreira de Arruda, Rinaldo Poncio Mendes

**Affiliations:** Faculdade de Medicina de Botucatu, UNESP - Univ Estadual Paulista, Botucatu, 18618-970 SP Brazil; Faculdade de Ciências, UNESP - Univ Estadual Paulista, Bauru, Departamento de Ciências Biológicas, Laboratório de Imunopatologia Experimental (LIPE), Bauru, 17047-001 SP Brazil

**Keywords:** Paracoccidioidomycosis, Monocyte subsets, Pulmonary fibrosis, Antifungal therapy

## Abstract

**Background:**

Paracoccidioidomycosis (PCM) is systemic mycosis caused by the thermal dimorphic fungus of genus *Paracoccidioides*, leading to either acute/subacute (AF) or chronic (CF) clinical forms. Numerous CF patients after treatment exhibit sequels, such as pulmonary and adrenal fibrosis. Monocytes are cells that are involved in the inflammatory response during active infection as well as in the fibrogenesis. These cells comprise a heterogeneous population with distinct phenotypic and functional activities. The scope of this study was to identify changes regarding functional and phenotypical aspects in monocytes comparing CF PCM patients on antifungal treatment versus non-treated patients (PMC-p).

**Methods:**

Twenty-three CF PCM composed of 11 non-treated patients (NTG) and 12 patients in apparent cure (ACG) were studied. Sixteen healthy individuals were used as control group (CG). Monocyte subsets were determined by immunophenotyping based on CD14 and CD16 expression. Cellular function was measured *in vitro* with and without stimulation with lipopolysaccharide (LPS) and *P. brasiliensis* exoantigen (PbAg) for 24 hours. Independent samples were compared using unpaired *t* tests, dependent samples were analyzed by paired t-test. Groups of more than two independent samples were analyzed using an ANOVA, with Tukey's post-test. Significance was set up at p <0.05.

**Results:**

Our results showed high counts of peripheral blood CD14^+^CD16^+^ and CD14^+^CD16^++^ monocytes in untreated PCM-p accompanied by intense production of pro-inflammatory cytokines (IL-1β and TNF-α) and profibrotic growth factors (TGF-β1 and bFGF) by monocytes challenged with *P. brasiliensis* antigens. After the introduction of antifungal therapy, the counts of CD14^+^CD16^+^ cells returned to baseline while CD14^+^CD16^++^ counts remained high. Interestingly, counts of CD14^+^CD16^++^ monocytes remained elevated even 52 ± 7 months after successful antifungal treatment. Furthermore, the ACG-patients showed preserved pro-inflammatory activity in the presence of specific antigen stimuli and high spontaneous production of TNF-α by monocytes.

**Conclusions:**

Infection with *Paracoccidioides* leads to initiation of a specific proinflammatory response by monocytes of PCM-p during active disease and in the apparent cure. A profibrotic profile by monocytes was observed only at admission. Furthermore, PCM-p with apparent cure showed high spontaneous production of TNF-α and high counts of CD14^+^CD16^++^ monocytes, probably induced by hypoxia duo to fibrotic sequelae.

**Electronic supplementary material:**

The online version of this article (doi:10.1186/s12879-014-0552-x) contains supplementary material, which is available to authorized users.

## Background

Paracoccidioidomycosis (PCM) is a systemic and granulomatous mycosis caused by thermally dimorphic fungi of the *Paracoccidioides brasiliensis* complex and *P. lutzii*[1]-[3] and is characterized by antigen-dependent immunosuppression [4]. The active disease presents two main clinical forms: the acute/subacute form (AF) and the chronic form (CF). PCM patients (PCM-p) with the AF are typically young and normally show a short duration of the symptomatology (median of two months), exhibiting clinical manifestations that involve organs rich in the mononuclear phagocytic system (e.g. bone marrow, liver, spleen). CF usually affects adult males who present a long duration of the symptomatology (>6 months) and exhibit predominant pulmonary and mucocutaneous involvement [5]. After treatment, numerous CF patients present sequelae, especially pulmonary fibrosis and emphysema, and Addison's syndrome [6].

As observed in other diseases caused by intracellular pathogens, the control of PCM depends on an effective and protective *Paracoccidioides*-specific host cellular immune response [7]. Although the adaptive immune response toward PCM is better characterized, immunity against the genus *Paracoccidioides* is complex and involves other immune cell subsets, including mononuclear phagocytes [7]. The immunophenotyping and functions under inflammatory and steady-state conditions of murine and human monocyte subsets have been investigated [8], and several studies have shown that monocyte subsets play different roles in the innate immune response during infectious processes and fibrogenesis [9],[10].

In humans, monocyte subsets can be identified by the expression of the surface molecules CD14 and CD16 [11]. CD14^+^CD16^−^ monocytes are termed classical monocytes and represent approximately 90-95% of human monocytes. These cells express high amounts of CCR1 and CCR2 and are characterized by moderate levels of the fractalkine receptor CX3CR1 and low HLA-DR. Furthermore, these cells exhibit intense phagocytic activity, produce high amounts of IL-10 upon lipopolysaccharide (LPS) stimulation and are the main precursors of tissue macrophages (reviewed in Zimmermann *et al.*[12]). Intermediate (CD14^+^CD16^+^) and non-classical or inflammatory (CD14^+^CD16^++^) subsets produce large amounts of proinflammatory mediators and are up-regulated in many inflammatory disorders, such as rheumatoid arthritis, atherosclerosis, bacterial sepsis and various hepatic diseases. Intermediate monocytes display high levels of HLA-DR, the mannose receptor and CCR5, and inflammatory monocytes exhibit high expression of CX3CR1 [12].

In PCM, the recovery of the immune response is a crucial step for considering successful the antifungal therapy. However, few reports have addressed immunological studies during the follow-up of PCM-p. Because interference with monocyte subset distribution and activation may be involved in PCM, we evaluated the phenotypic and functional aspects of the monocytes in non-treated CF PCM-p, as well as in patients that with apparent cure.

## Methods

### Patients

Twenty-three CF PCM patients (PCM-p) from the Tropical Diseases Ward and Outpatient Clinic for Paracoccidioidomycosis at the University Hospital, Faculdade de Medicina de Botucatu (FMB), UNESP–Univ. Estadual Paulista, Botucatu, SP, Brazil, were studied. Cases with clinical manifestations that were compatible with PCM were considered either confirmed or probable [13]. Cases were considered confirmed when the typical *Paracoccidioides* genus yeast forms were identified in the clinical specimens and probable when only serum-specific antibodies were detected using a double agar gel immunodiffusion test (DID). All patients exhibited pulmonary involvement and were classified as having clinical CF. Patients who exhibited neoplasia, inflammation, infectious diseases or pregnancy were not enrolled.

### Ethics statement

This study was approved by the Research Ethics Committee of FMB-UNESP (#3145/2009). Written informed consent to participate and to publish the data was obtained from all participants. In this study IRB was signed by all the adult patients. We had no IRB signed by the closest relative or the legal representative.

### Experimental design

PCM-p were categorized into two groups: the non-treated group (NTG), consisting of 11 newly diagnosed patients, and the apparent cure group (ACG), consisting of 12 PCM-p who did not show any signs or symptoms and had a normal erythrocyte sedimentation rate (ESR), negative serology, and at least 2 full years of non-treatment after complete antifungal therapy [6]. The homogeneity of the groups was determined based on sex, age (years), clinical form and degree of severity, specific antibody serum levels and information on antifungal treatment (Table 1). Sixteen age- and sex-matched healthy individuals were selected among blood donors from the same geographical area to form the CG.

**Table 1:** **Clinical characterization of the patients**

Patients	Sex	Age^*^	Degree of severity^*^	DID (1: ) - Admission^*^	Group	Antifungal	Length of treatment (months)^**^	Length after treatment (months)^**^
P1	M	60	Moderate	16	NTG	-	-	-
P2	M	53	Severe	128	NTG	-	-	-
P3	M	59	Moderate	32	NTG	-	-	-
P4	F	47	Moderate	64	NTG	-	-	-
P5	M	44	Moderate	64	NTG	-	-	-
P6	M	51	Mild	NR	NTG	-	-	-
P7	M	64	Moderate	32	NTG	-	-	-
P8	M	59	Moderate	32	NTG	-	-	-
P9	M	52	Moderate	8	NTG	-	-	-
P10	M	47	Moderate	32	NTG	-	-	-
P11	M	37	Moderate	4	NTG	-	-	-
P12	M	56	Moderate	16	ACG	CMX	25	28
P13	M	39	Severe	64	ACG	CMX	173	37
P14	M	60	Mild	16	ACG	CMX	97	45
P15	M	56	Moderate	16	ACG	CMX	26	3
P16	M	50	Moderate	512	ACG	CMX	53	62
P17	M	42	Moderate	8	ACG	CMX	28	83
P18	M	42	Moderate	NR	ACG	ITC	31	69
P19	M	39	Moderate	2	ACG	ITC	32	27
P20	M	40	Severe	128	ACG	CMX/ITC	33^(CMX)^/49^(ITC)^	89
P21	M	42	Severe	64	ACG	CMX/ITC	6^(CMX)^/131^(ITC)^	53
P22	M	51	Severe	128	ACG	CMX/ITC	44^(CMX)^/12^(ITC)^	60
P23	M	53	Severe	64	ACG	CMX	84	65

DID = double agar gel immunodiffusion test; NR = non-reagent; NTG = non-treated group; ACG = apparent cure group; CMX = cotrimoxazole; ITC = Itraconazole.

*Homogeneity (NTG x ACG):

Age: NTG =52 (37–64); ACG = 46 (39-60); *p* =0.18 (Mann–Whitney U test).

Degree of severity: NTG = ACG; *p* =0.31 (Fisher's exact test).

DID admission: NTG =32 (NR-1:128); ACG =32 (NR-1:512); *p* =0.66 (Mann-Whitney U test).

**Length of treatment (Mean ± SEM): 68 ± 14; Length after treatment (Mean ± SEM): 52 ± 7.

### Determination of the peripheral blood monocyte subsets

Venous blood was collected in Vacutainer tubes (BD, Becton Dickinson, Franklin Lakes, NJ, USA) containing EDTA anticoagulant. Whole blood (100 μl) was added into polystyrene tubes containing the following monoclonal antibodies: phycoerythrin (PE)-conjugated mouse IgG1 anti-human CD14, clone HCD14; peridinin chlorophyll protein complex (PerCP)-conjugated mouse IgG1 anti-human CD16, clone 3G8; and allophycocyanin (APC)-conjugated mouse IgG1 anti-human CD45, clone HI30, all of which were purchased from BioLegend (San Diego, CA, USA). The tubes were incubated for 20 min at 4°C and again after FACS lysing solution was added. The cells were washed with BD Pharmingen™ stain buffer and analyzed using a FACSCalibur (BD). The data were analyzed using the FlowJo software (Tree Star Inc, USA). The cell counts were calculated based on peripheral leucocyte count (WBC).

### Monocyte culture

Twenty milliliters of venous blood were collected in Vacutainer® tubes (BD) containing heparin anticoagulant. Mononuclear cells were separated using a Ficoll-Hypaque (Sigma, St Louis, MO, USA) density gradient and suspended in 1.0 ml of RPMI-1640 medium containing L-glutamine and 25 mM HEPES buffer (Gibco, Grand Island, NY, USA) supplemented with 10% fetal calf serum (Gibco), penicillin (100 UI/ml) and streptomycin (100 μg/ml) (Gibco). The cell concentration was adjusted to 2.0 × 10^6^ mononuclear phagocytes ml^-1^, as judged by the uptake of 0.02% neutral red. The mononuclear cells were placed in 96-well, flat-bottomed microtiter plates (Costar, Cambridge, MA, USA) and incubated for 2 hours at 37°C and 5% CO_2_ in a humidified chamber to allow the monocytes to adhere and spread. Non-adherent cells were removed by washing the wells 3 times with RPMI-1640 medium, and the remaining adherent cells (>90% mononuclear phagocytes as assessed by the CD14 cell-surface expression) were used for experiments. The adherent monocytes were cultured at 37°C and 5% CO_2_ in supplemented RPMI-1640 in the presence of medium only or optimal concentrations of LPS (10 μg ml^-1^, Sigma) and *P. brasiliensis* exoantigen (20 μg ml^-1^, Pb113 strain). After 24 hours, the cell-free supernatants were harvested and stored at -80°C pending cytokine analysis.

### Quantification of cytokines

The cytokines TGF-β1, CCL3/MIP-1α, VEGF and bFGF were quantified using ELISA with the Cytokine Duo-Set Kit (R&D Systems, Minneapolis, USA). IL-1β, IL-6, IL-10, TNF-α and IL-12p70 were quantified using flow cytometry and the Human Inflammation Kit, BD™, Cytometric Bead Array (BD).

### Statistical analysis

Statistical tests were performed using the GraphPad v.5.00 software (GraphPad Software Inc, San Diego, CA, USA), and significance was set up at *p* ≤0.05 for all of the analyses. The homogeneity of the groups NTG and ACG was determined by Mann-Whitney U test and Fisher's exact test. Comparisons of two independent samples were performed using unpaired *t* test, two dependent samples using *t*-paired test, and comparison of more than two independent samples were performed using an ANOVA, with Tukey's post-test [14].

## Results

### Distribution of the blood peripheral monocyte subsets of PCM-p before antifungal treatment, during the antifungal treatment and after apparent cure

**Figure 1:**
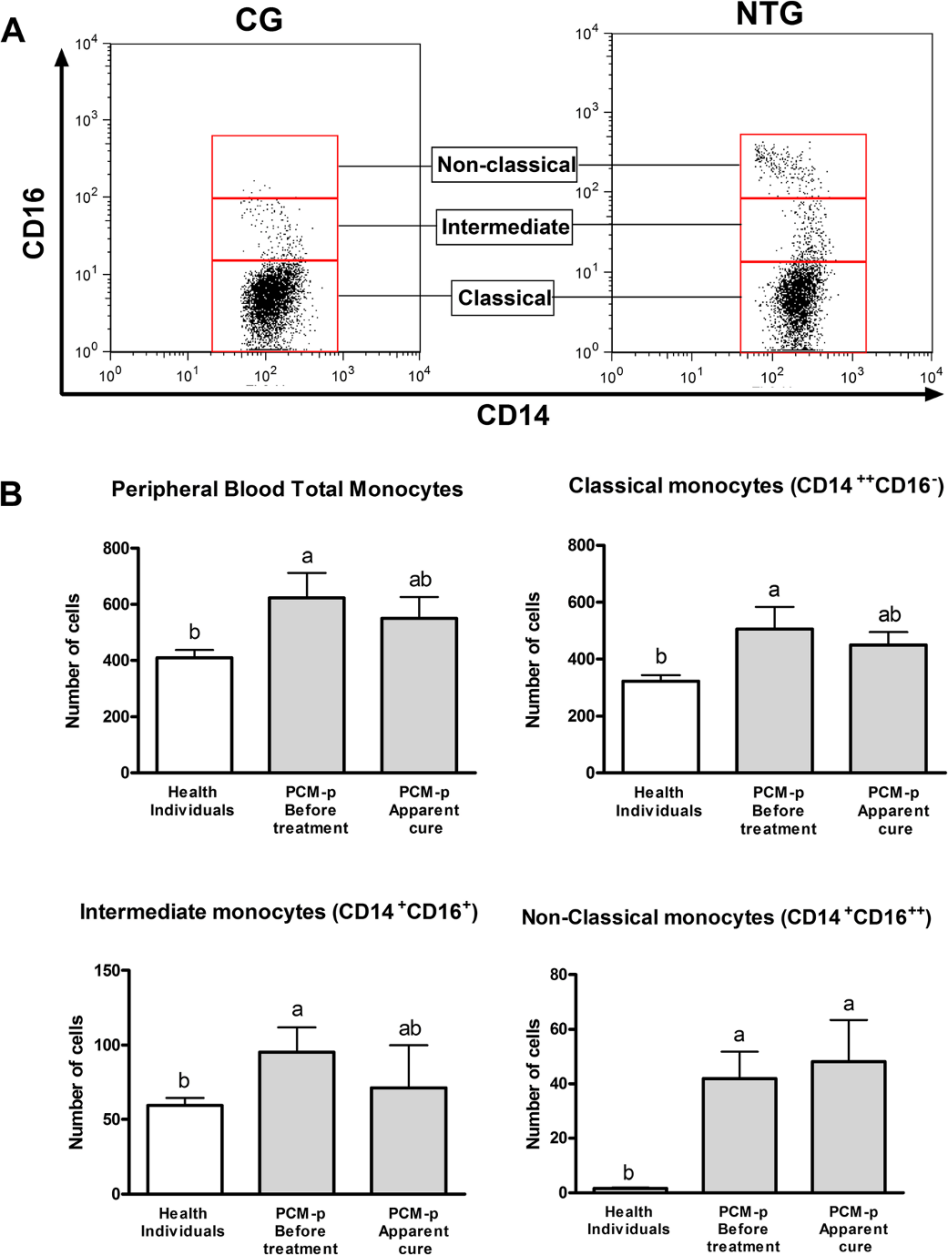
**Distribution of peripheral blood monocyte subsets. A**. Dot plots showing the gate strategies used to identify the monocyte subsets (from CD45^+^CD14^+^) of healthy individuals and untreated patients. **B**. Distribution of the total peripheral blood monocytes and their subsets in healthy individuals (CG, n =16), patients with PCM (PCM-p) before antifungal treatment (NTG, n =9) and PCM-p with apparent cure (ACG, n =12). Values are expressed as the mean ± standard deviation (cell counts of total peripheral leukocytes - CD45^+^ cells) and comparisons were performed by ANOVA with Tukey's post-test. Different letters indicate significant differences among the groups (*p* ≤0.05).

Although in both groups of PCM-p the total monocyte count (CD45^+^CD14^+^) was in the normal clinical range compared to healthy controls, the NTG group still showed significantly elevated CD45^+^CD14^+^ cell counts (Figure 1C). In this group, we also observed high counts of CD14^+^CD16^+^ and CD14^+^CD16^++^ (Figure 1A, B and C). Five PCM-p from NTG were also evaluated after the introduction of antifungal therapy; we observed a significant decrease in CD45^+^CD14^+^ cells and the counts of CD14^+^CD16^++^ cells remained high (Table 2). PCM-p from ACG showed high counts of CD14^+^CD16^++^ (Figure 1C).

**Table 2:** **Distribution of monocyte subsets of PCM-p during the follow-up**

ID patient	Before Treatment				During the treatment				
	CD45^+^CD14^+^	CD14^+^CD16^-^	CD14^+^CD16^+^	CD14 + CD16^++^	CD45^+^CD14^+^	CD14^+^CD16^-^	CD14^+^CD16^+^	CD14^+^CD16^++^	Length of treatment (months)
**P3**	348	327	16	5	331	307	6	18	17 (ITC)
**P6**	933	716	158	58	221	430	16	18	20 (CMX)
**P7**	1164	959	118	88	776	610	4	23	20 (ITC)
**P8**	457	346	82	30	214	290	6	10	20 (CMX)
**P9**	511	350	141	20	273	360	2	15	17 (ITC)
**Mean ± SEM**	**682.6 ± 156.0** ^**A**^	**539.6 ± 127.6**	**103.0 ± 25.2** ^**A**^	**40.2 ± 14.7**	**363.0 ± 105.4** ^**B**^	**399.4 ± 58.0**	**6.8 ± 2.4** ^**B**^	**16.8 ± 2.1**	**18.8 ± 1.4**

Different letters indicate significant differences between the groups (*p* ≤0.05, *t*-paired test; A >B).

Values expressed as cell counts/μl.

### Production of inflammatory mediators/cytokines by monocytes from PCM-p before treatment and after apparent cure

We determined the concentrations of IL-1β, IL-6, TNF-α, IL-10, IL-12p70, CCL3/MIP-1α, TGF-β1, FGF-b and VEGF in the supernatants of monocyte cell cultures in the presence or absence of LPS-nonspecific and *P. brasiliensis* antigen-specific stimuli. Among the mediators analyzed in the cell-free supernatant, VEGF and IL-12p70 had levels that were below the detection limits.

**Figure 2:**
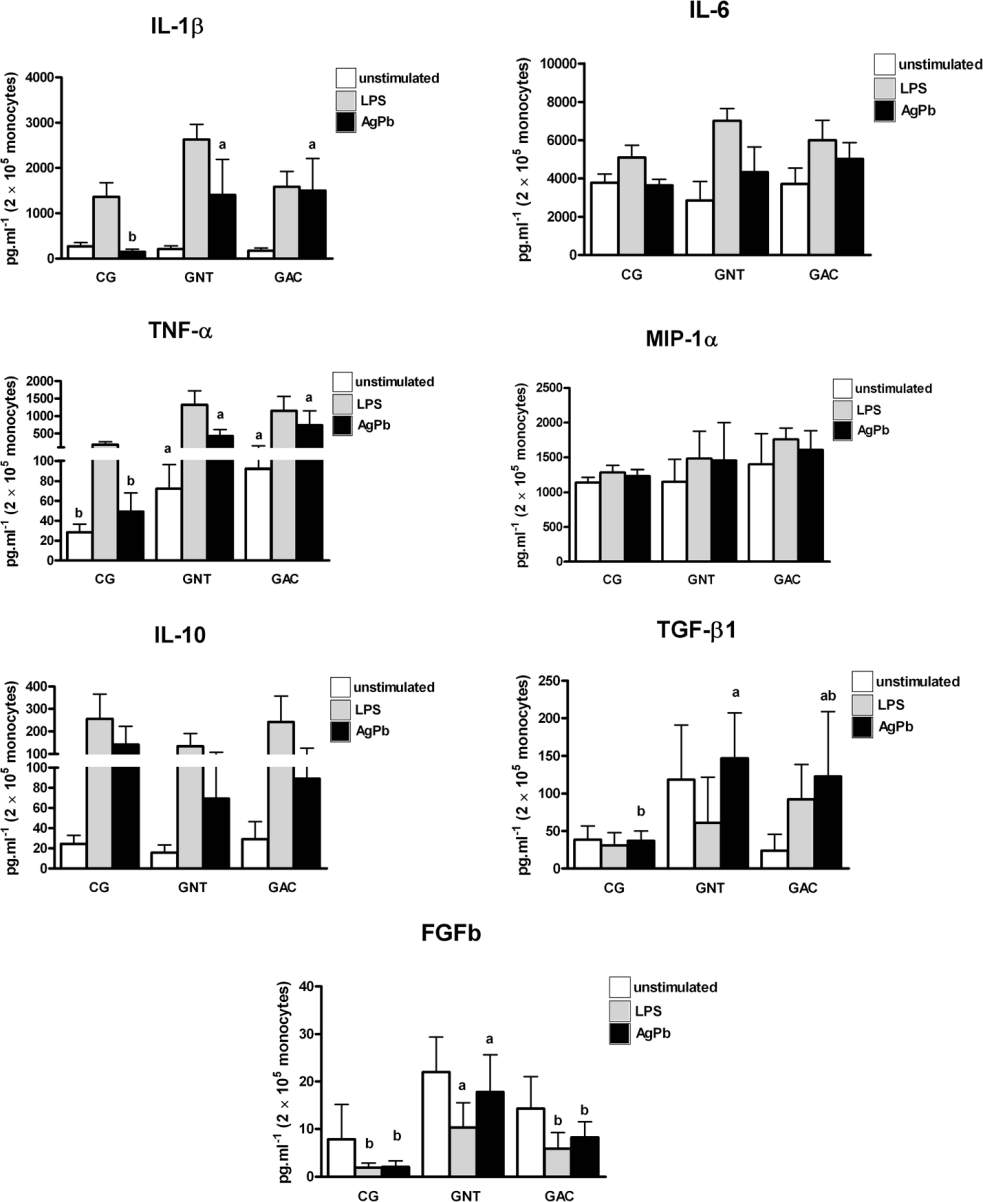
**Determination of IL-1β, IL-6, TNF-α, MIP-1α, IL-10, TGF-β1 and FGFb levels in the cell-free supernatant obtained from monocytes from healthy subjects (CG, n =6), PCM-p before treatment (NTG, n =7) and PCM-p with apparent cure (ACG, n =8).** The cells were cultured in the absence (spontaneous production – unstimulated) or presence of lipopolysaccharide - LPS and *P. brasiliensis* antigen - AgPb (stimulated production). Values are expressed as the mean ± standard deviation and comparisons were performed by ANOVA with Tukey's post-test. Different letters indicate significant differences among the groups (*p* ≤0.05).

Independent of the stimuli, no differences in the production of IL-6, IL-10 and MIP-1α were observed among the groups (Figure 2). The production of IL-1β and TNF-α after specific-antigen challenge was higher in PCM-p regardless of treatment compared with the CG. Moreover, the spontaneous production of TNF-α was higher in both groups of patients than in the CG (Figure 2).

The specific-stimulus production of TGF-β1 was higher in non-treated patients than normal subjects and similar to PCM-p with apparent cure (Figure 2). Similarly, the specific and non-specific stimulus production of bFGF was higher in non-treated patients than normal subjects and similar to PCM-p with apparent cure (Figure 2).

## Discussion

Some aspects should be considered regarding the immunological evaluations of CF PCM-p. First, the CF is usually caused by reactivation of latent foci that were maintained for an unknown amount of time, potentially as long as 30 years [15]; thus, the host has previously organized an adaptive immune response against the genus *Paracoccidioides* that was efficacious for some time. Indeed, the prevalence of healthy subjects from endemic areas who have a positive intradermal skin test to paracoccidioidin and negative test for histoplasmin can reach 50% [16],[17]. Second, due to the long duration of symptomatology of >6 months, the clinical and immunological evaluations performed at admission occur in PCM-p who have responded to the paracoccidioidal antigens but without success because they show progression of the signs and symptoms [5]. In addition, the inflammatory response is intense but inefficacious [18]-[21]. Third, the lungs are the entry route for the fungus and one of the main target organs in the CF [22]. In this organ, peri-bronchial pulmonary lesions of the epithelioid granulomatous type are observed, followed by confluence and nodule formation, occasionally with central necrosis, which can drain its contents and form cavities [23],[24]. Furthermore, intense collagen I and III (reticulin fibers) deposition around the granuloma that interconnects neighboring structures, such as bronchi and blood vessels, occurs. In addition, reticulin proliferation is observed in the alveolar septae in non-granulomatous areas [25]. It is important to highlight that upon admission, CF patients typically present all of these pulmonary alterations, as identified by several biopsies and necroscopic studies [24],[26].

Here, we showed for the first time high counts of peripheral blood CD14^+^CD16^+^ intermediate as well as CD14^+^CD16^++^ non-classic monocytes in untreated PCM-p. Studies on infectious processes, such as Gram-negative bacteremia, have shown an increased number of monocytes CD16^+^ that normalizes after efficacious treatment [27]. In the present study, we observed that after the introduction of the antifungal therapy, the count of total monocytes and CD14^+^CD16+ cells decreased. All groups of PCM-p exhibited high counts of CD14^+^CD16^++^.

In addition to infectious processes, a recent report showed a higher frequency of intrahepatic intermediate monocytes in patients with chronic inflammatory and fibrotic liver diseases [28]. According to the authors, the intermediate subset can modulate liver fibrogenesis. Although we did not separately evaluate the functions of the monocyte subsets, the high percentage of CD16^+^ monocytes in untreated CF PCM-p could be related to two typical morpho-functional aspects of these patients: an intense inflammatory response and pro-fibrotic activity. We observed high production of pro-inflammatory cytokines (IL1-β and TNF-α) and pro-fibrotic growth factors (TGF-β1 and bFGF). This profile was similar to that observed in previous studies that evaluated several pro-inflammatory cytokines and TGF-β1 [18],[21]. Considering that TGF-β1 is a pleiotropic cytokine that exhibits a central role in fibrogenesis [29], as well as potent anti-inflammatory/regulatory activity [30]_,_ the bFGF dosage enhances early fibrogenesis in CF PCM-p.

In addition, we observed that some patients showed a normal distribution of monocyte subsets and/or levels of TGF-β1 and bFGF. This finding could be associated with clinical observations of different degrees of fibrosis upon patient admission. Our group recently developed an algorithmic method to quantify pulmonary fibrosis and emphysema [31], and a study to correlate the different degrees of fibrosis and with the immune response is ongoing.

Herein, we also demonstrate for the first time that *P. brasiliensis* antigens significantly enhance the production of IL1-β, TNF-α, TGF-β1 and bFGF by peripheral blood monocytes of PCM-p, suggesting that fungal metabolites play an important role in the activation of these cells. Considering that monocytes are precursors for various tissue macrophages and dendritic cells and secrete several immune mediators in the pulmonary milieu during inflammation, it is possible that the recruitment of specific-stimulated monocytes into paracoccidioidal lesions could contribute to deleterious inflammatory tissue damage and trigger fibroblast and fibrocyte activation [32]. This hypothesis is supported by several studies that have demonstrated an array of strategies used by *P. brasiliensis* or its metabolites to manipulate host cells to survive and propagate (reviewed in Mendes-Giannini *et al.*[33]). Thus, we believe that our findings should open new perspectives for investigation, especially related to fibrosis in PCM-p.

We also investigated for the first time the functional aspects of the peripheral blood monocytes of PCM-p after treatment, i.e., with apparent cure. We found high levels of TNF-α and IL-1β in antigen-stimulated cell culture supernatants, which suggests preserved pro-inflammatory activity of the PCM-p monocytes when stimulated by paracoccidioidal antigens. In addition, we observed higher counts of CD16^++^ monocytes and high levels of spontaneous TNF-α release by monocytes from these patients. Considering that the pulmonary sequelae observed in CF PCM-p typically become worse after antifungal therapy is initiated [6],[34], it is possible that the higher levels of TNF-α are due to hypoxemia [35].

## Conclusions

In summary, the present study showed that untreated PCM-p exhibit intense production of pro-inflammatory and pro-fibrotic mediators by monocytes challenged with *P. brasiliensis* antigens. They also exhibit high counts of CD14^+^CD16^+^ and CD14^+^CD16^++^ monocytes. After the introduction of antifungal therapy, the counts of CD14^+^CD16^+^ returned to baseline. After complete treatment, monocytes from PCM-p showed preserved pro-inflammatory activity against specific stimuli, high spontaneous production of TNF-α and high counts of CD14^+^CD16^++^. Although the role of these cells is complex, we suggest that during the active disease, these cells could act both in the proinflammatory response as wells as in the fribrogenesis; and after the treatment, the findings seems to be a consequence of hypoxemia due to pulmonary fibrosis.

## Authors’ original submitted files for images

Below are the links to the authors’ original submitted files for images. Authors’ original file for figure 1 Authors’ original file for figure 2

## Abbreviations

ACG: Apparent cure group
AF: Acute/subacute form
ANOVA: Analysis of variance
APC: Allophycocyanin
bFGF: Basic fibroblast growth factor
CCR: Chemokine receptors of the of the CC subfamily
CF: Chronic form
CG: Control group
CX3CR: Chemokine receptors of the of the CXC subfamily
DID: Double agar gel immunodiffusion test
EDTA: Ethylenediamine tetraacetic acid
ELISA: Enzyme-linked immuno sorbent assay
ESR: Erythrocyte sedimentation rate
FACS: Fluorescence-activated cell sorting
HEPES: Hydroxyethyl piperazineethanesulfonic acid
HLA: Human leukocyte antigen
IgG: Immunoglobulin G
IL: Interleukin
LPS: Lipopolysaccharide
MIP: Macrophage inflammatory protein
NTG: Non-treated group
PbAg: *P. brasiliensis* exoantigen
PCM: Paracoccidioidomycosis
PMC-p: Paracoccidioidomycosis patients
PE: Phycoerythrin
PerCP: Peridinin chlorophyll protein complex
TGF: Transforming growth factor
TNF: Tumor necrosis factor
VEGF: Endothelial growth factor
