# Supplementary figures and images for: Phenotypic and functional evaluations of peripheral blood monocytes from chronic-form paracoccidioidomycosis patients before and after treatment
Source: BMC Infect Dis. 2014 Oct 16;14:552. doi: 10.1186/s12879-014-0552-x (PMC4201701; doi:10.1186/s12879-014-0552-x)

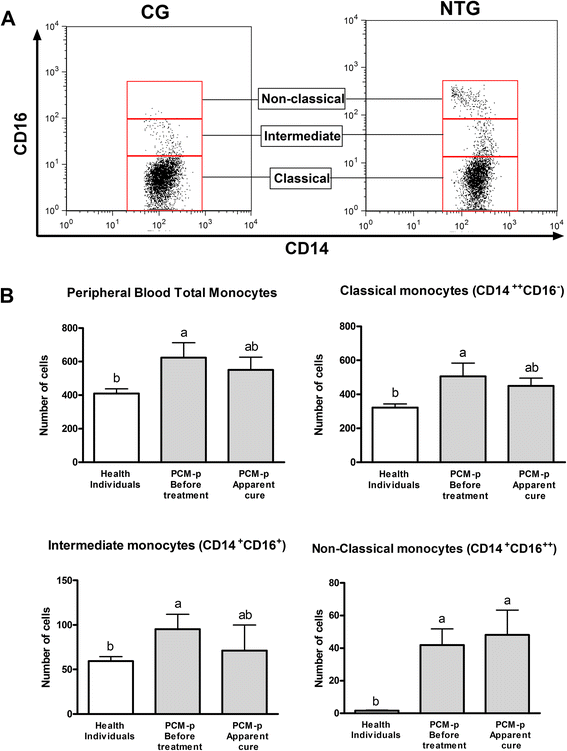

Supplement: Supplementary file 1 — Authors’ original file for figure 1 [file 12879_2014_552_MOESM1_ESM.gif]

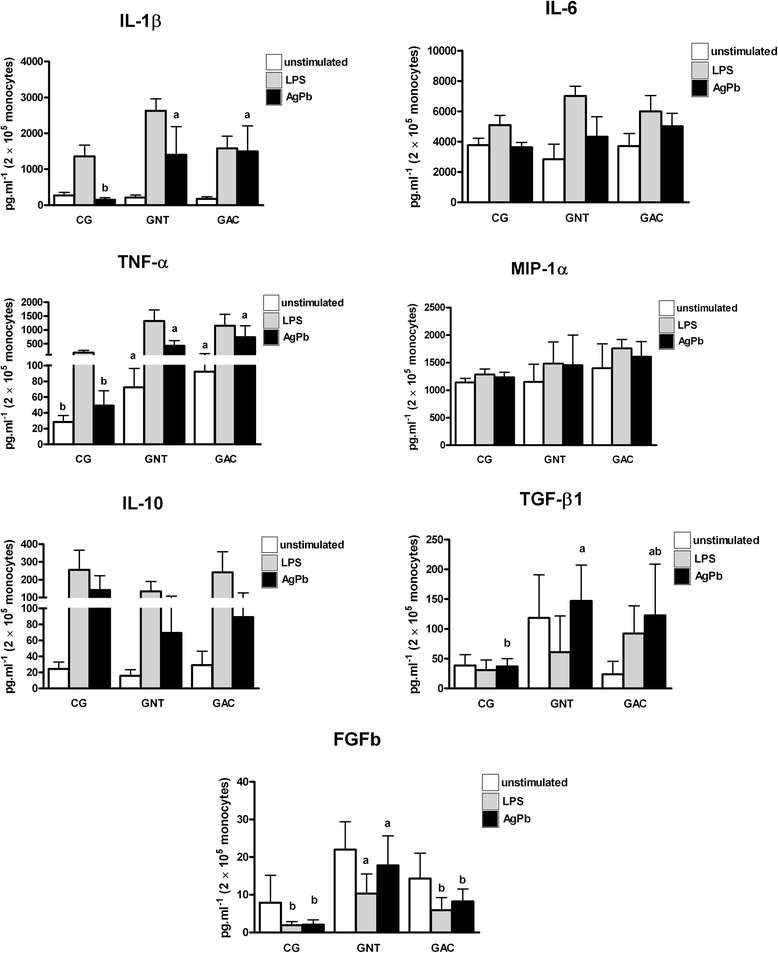

Supplement: Supplementary file 2 — Authors’ original file for figure 2 [file 12879_2014_552_MOESM2_ESM.gif]
